# Uterine contractility changes in a perfused swine uterus model induced by local anesthetics procaine, lidocaine, and ropivacaine

**DOI:** 10.1371/journal.pone.0206053

**Published:** 2018-12-06

**Authors:** Fabian Weinschenk, Ralf Dittrich, Andreas Müller, Laura Lotz, Matthias W. Beckmann, Stefan W. Weinschenk

**Affiliations:** 1 Department of Plastic, Reconstructive, Hand-, and Burn Surgery, StKM Klinikum Bogenhausen, Academic Teaching Hospital Technical University, Munich, Germany; 2 Department of Obstetrics and Gynecology, University Hospital Erlangen, Erlangen, Germany; 3 Department of Obstetrics and Gynecology, Städtisches Klinikum Karlsruhe, Karlsruhe, Germany; 4 Department of Gynecological Endocrinology and Fertility Disorders, University of Heidelberg, Heidelberg, Germany; 5 Outpatient OB/GYN Practice Weinschenk, Scherer & Colleagues, Karlsruhe, Germany; 6 Heidelberg University Neural Therapy Education and Research Group (The HUNTER Group), Heidelberg, Germany; University of Bari, ITALY

## Abstract

**Background:**

Local anesthetics (LAs) are increasingly used as therapeutics due to their multiple molecular effects. They may be potential agents also in gynecology and reproductive medicine. The objective of this study was to investigate the contractility response of the perfused swine uterus to different concentrations of the LAs procaine, lidocaine, and ropivacaine.

**Methods and findings:**

In an extracorporeal perfusion model with fresh swine uteri, effects of administered boli of these three LAs in concentrations of 0.1 mg/mL, 0.5 mg/mL and 1.0 mg/mL on uterine contractility and peristalsis were assessed using an intrauterine double-chip micro-catheter. A dose-dependent increase in intrauterine pressure (IUP) in the isthmus and corpus uteri was observed after the administration of the ester-LA procaine 0.1, 0.5, and 1.0%, which was not seen with lower concentrations, or buffer solution. An increase-decrease curve was found after increasing concentrations of the amide-LA lidocaine and ropivacaine, with an IUP plateau with 0.1 and 0.5%, and a decrease with 1% (p<0.01). All reactions were seen in both the isthmus and corpus uteri. The difference of the contractility pattern between ester- and amide-LA at 1% concentration was significant.

**Conclusion:**

LAs dose-dependently modulate contractility in non-pregnant swine uteri. The amid-LAs lidocaine and ropivacaine reduce contractility in higher concentrations and may be used as therapeutics in disorders with increased uterine contractility, as dysmenorrhoea, endometriosis, and infertility. The multiple molecular effects of LAs may explain these effects. This in-vitro pilot study in vitro provides initial data for designing further studies to transfer the results onto humans.

## Introduction

Local anesthetics (LAs) are commonly used in anesthesiology for analgesia. Recently, they were revealed to have further clinical effects by their so-called “alternative mechanisms” [1] in different fields of medicine. Accordingly, it is interesting to study their effects on the reproductive system as well.

The mechanism of analgesic effects of LAs has been known since the 1950s [2]. LAs reversibly block voltage-sensitive sodium channels [3]. However, some so-called “alternative effects” of LAs could not be explained with the sodium-ion receptor affinity but could be related to other receptors. For instance, anti-inflammatory mechanisms have been assigned to effects on the alpha subunit of G proteins [1]. Furthermore, LAs have been reported to be antithrombotic [4], bacteriostatic [5], and antiviral [6]. Also, in clinical settings, studies showed LA effects beyond their analgesic properties, as improvement of postoperative bowel activity after abdominal surgery [7–8]. In gynecology, positive effects were found, for instance, in dysmenorrhea [9], endometriosis, and perturbation [9–10]. In reproductive medicine, however, there is only little data on therapeutic effects of LAs. For instance, Lidocaine inhibits sperm phagocytosis and thus increases the gestation rate by 4.5 times [11].

The primary goal of the present pilot study was to examine the effects of LAs on uterine contractility. Uterine contractility is the major feature of the organ concerning most functions. Contractility disturbances have been shown in disorders as endometriosis, dysmenorrhea [12], and infertility [9]. Therefore, finding new substances to normalize pathological contractility is crucial for treating these disorders, either by direct effects on the myometrium or by influencing the autonomic innervation of the organ [13]. LAs are promising substances to fulfill this purpose.

In order to investigate the effects of LA on the reproductive system, we conducted contractility experiments in an in-vitro model. Contractility examinations of the perfused swine uterus are a suitable in-vitro measure that allows the examination of pharmacological substances on uterus contractility [14]. The advantage of working on the entire organ rather than on isolated muscle strips is the better transferability to future clinical tests in humans.

Secondary aims of the study were to determine if different LAs have different effects on uterine contractility. LAs are categorized in two groups according to their molecular structure. LAs of the ester type, such as procaine and tetracaine, are degraded at the site of application in humans, catalyzed by the ubiquitous cholinesterase producing defined metabolites. LAs of the amide type (lidocaine, bupivacaine, ropivacaine and many more) remain unmodified and are excreted through the kidney, or are transported to the liver where they are metabolized [15]. We hypothesize that the molecular structure of LAs influences their effect on contractility.

Third, we sought to determine if there were dose-dependent differences in contractility changes by LAs. Therefore, we choose different concentrations that are commonly used in clinical practice.

Thus, if LAs were found to change the uterine contractility, we would be able to design clinical studies of therapeutic effects of LAs in gynecology, obstetrics, and reproductive medicine.

## Material and methods

### Swine uteri

**Fig 1:**
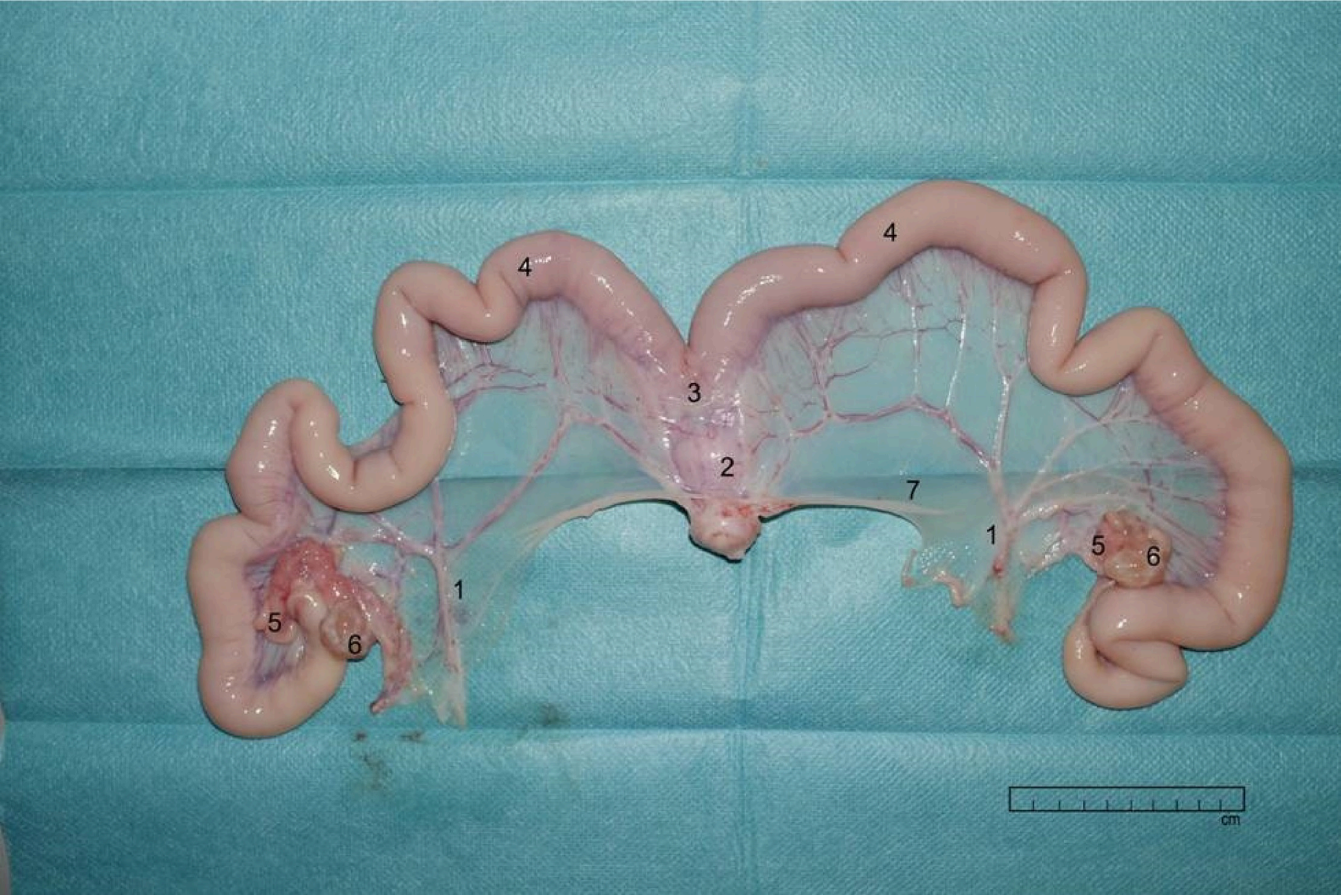
Prepared swine uterus before probe insertion. 1: uterine artery, 2: cervix, 3: uterine corpus, 4: Cornua uteri, 5: Tuba uterina, 6: ovaries, 7: connective tissue. Copyright: F. Weinschenk 2015.

The study was approved by the ethical committee of the University of Erlangen-Nuremberg. Swine uteri were collected from the abattoir in Erlangen, immediately after death by electric shock (1.5 A, 400 V, 4 sec). Uteri from healthy swine aged 7–18 months were selected for the experiments based on size, weight, general condition, and the integrity of the uterine arteries. The freshly slaughtered uteri were carefully chosen to obtain uteri in the diestrus phase of the oestral cycle. The cycle phase of swine uteri can be detected using the criteria edema and coloring, as described in [16]. After collection, each specimen was transferred immediately under constant temperature to the laboratory, prepared and placed into a 37°C organ bath of 1L Krebs-Ringer buffer (KRB) solution within 20 minutes, see Fig 1.

### Modified Krebs–Ringer buffer solution

Physiological modified Krebs–Ringer bicarbonate buffer solution (KRB) has been used by Dittrich et al. and Müller et al. since 2003 for experiments in the perfused swine uterus [14]. This modified form of KRB was previously described by Richter et al. for extracorporeal long-term perfusion of human uteri with recirculation of the perfusate [17]. The solution is capable of maintaining vitality and functionality of the tissue for a period of up to 24 hours [17–18]. Its composition ensures iso-osmotic ion concentration, physiological pH values (7.36–7.44), and a pK_s_ of 7.55. It maintains a colloid osmotic pressure of approximately 24 mmHg. Unlike Richter et al. [17], we did not use gentamicine, and in contrast to previous experiments performed in our group [19], we further omitted oxytocine, in order to avoid unpredictable influences of other substances on contractility. This KRB buffer solution was prepared in our institution following the manufacturer’s instructions (Sigma-Aldrich Ltd., Steinheim, Germany) and was used as organ bath and perfusion medium as well.

### Cannulation of the organ

**Fig 2:**
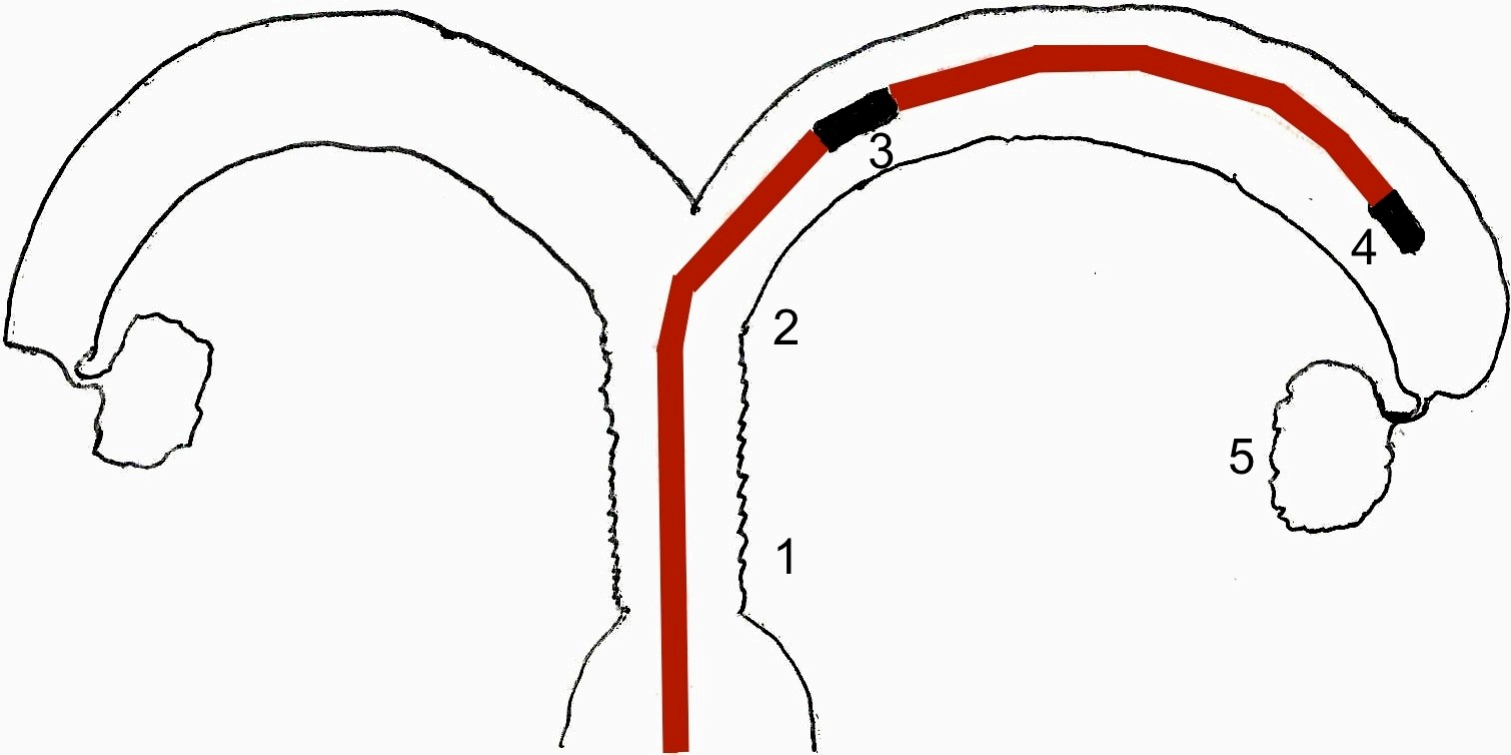
Intrauterine position of the probes. 1: Cervix, 2: uterine horn, 3: IUP2, 4: IUP1, 5: ovary. Copyright: F. Weinschenk, 2015.

The uterine arteries were released from the surrounding connective tissue in the broad ligament of the uterus bilaterally as far as the adventitia. Perfusion catheter insertion into the arteries was performed using 16-G Abbocaths, which were fixed in place using Vicryl 3–0 sutures (Ethicon Johnson & Johnson International Inc., Brussels). The procedure of cannulation has been described in detail by Dittrich et al [19] and others [17]. Correct positioning of the Abbocaths and patency of the uterine vascular system were confirmed by carefully rinsing the two arteries, each with 5 ml KRB solution. In addition, the tubes and the vascular plexus surrounding the ovary were ligated to prevent the perfusion solution from escaping at these points and to maintain better uterine perfusion pressure (see Fig 2).

### Perfusion system

The temperature of the KRB perfusion medium was kept constant at 36.5°C to 37.5°C with a temperature measurement probe (Raumedic; Rehau Ltd., Rehau, Germany) and oxygenated with carbogen (95% O_2_, 5% CO_2_) (Linde Inc., Frankfurt, Germany). In the first 14 experiments, due to a technical problem with the heater, the temperature was not 37°C, but room temperature. These experiments were excluded from data evaluation. The Abbocaths were connected with a silicon tube system. A roller pump (Heidolph Ltd., Kelheim, Germany) transported the medium at a constant flow rate (6 mL/min) into the organ’s arterial system. The solution flowed continuously through the uterus’s open vascular system into the organ bath, i.e., there was no recirculation of the medium into the arterial system.

### Control bolus

In order to prove that the bolus of nutrition solution did not change the contractility, we administered 1 ml plain KRB before each LA bolus. This was our “zero” (control) test. There was no change of uterine contractility (p = 0.153) before and after the injection of 1 ml KRB bolus (p < 0.05) with 37°C.

### Local anesthetics

Local anesthetics used in this survey are commercially available and were supplied by the hospital’s pharmacy (for details, see Table 1). The effective LA dosage was assessed in dose-finding studies performed before starting the experiments. Concentrations below 0.1% to 0.001% were found to not be different from the buffer solution.

**Table 1:** Pharmacologic properties of the three LAs used in this study.

	Trade name	Concentration	Manufacturer	Mole-cular structure	pKs	Protein binding	Lipid solubi-lity	0.1 mg/ml equals	0.5 mg/ml equals	1.0 mg/ml equals
**Procaine**	Novocain	1%	Steiger-wald	ester	8,05	6%	0,02	4.23 mmol/l	21.16 mmol/l	42.32 mmol/l
**Lidocaine**	Xylocain	1%	Com-bustin GmbH	amide	8,01	64%	2,9	4.27 mmol/l	21.34 mmol/l	42.67 mmol/l
**Ropiva-caine**	Narcain	1%	Jena-pharm	amide	8,1	95%	13,1	3.63 mmol/l	18.17 mmol/l	36.33 mmol/l

### Randomization and exclusion criteria

**Fig 3:**
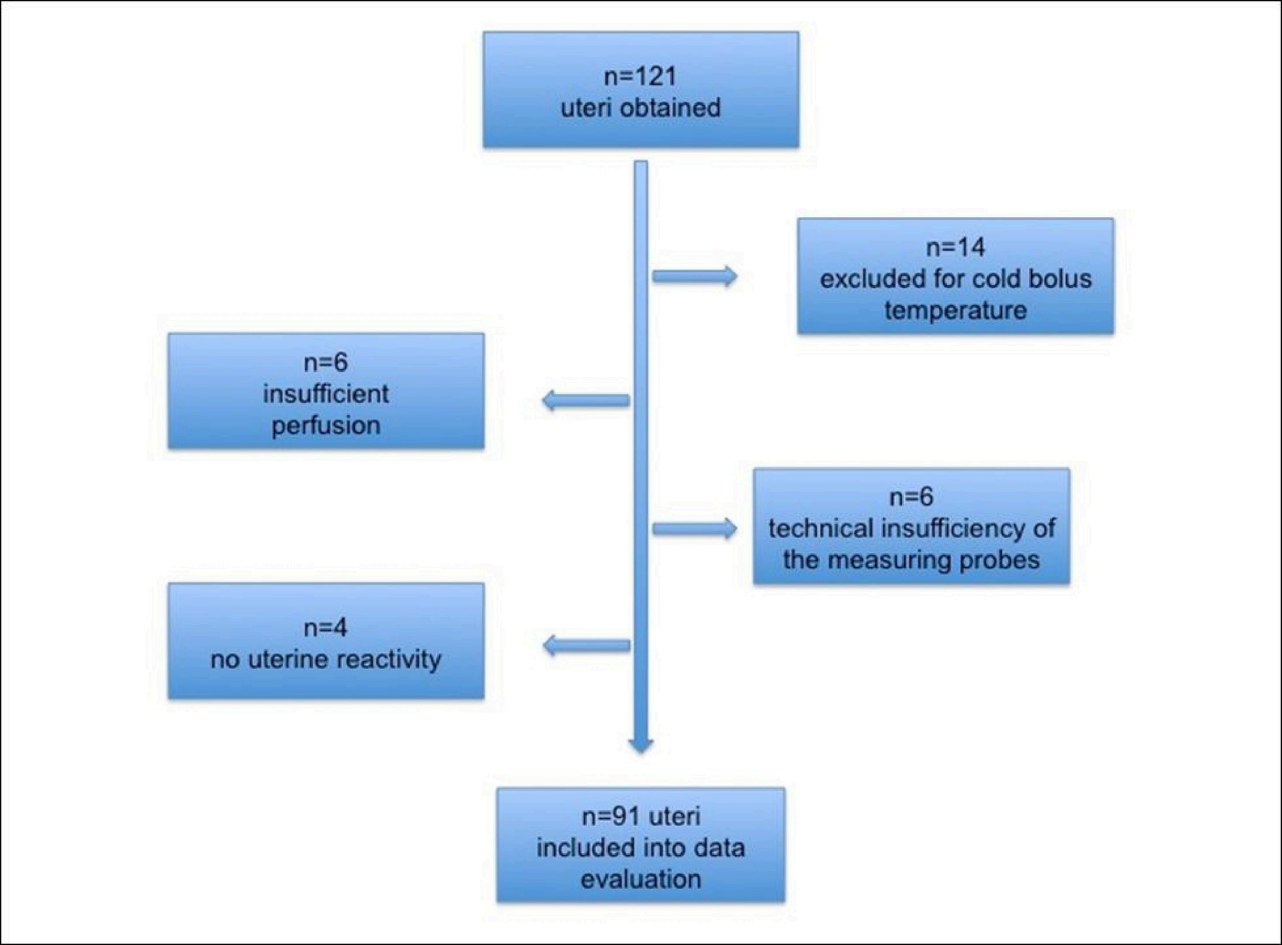
Specimen collection of swine uteri. 91 uteri of 121 (75.2%) could be included into data evaluation.

Uteri were randomly assigned to three groups. In all three groups of experiments, uteri were perfused with the modified KRB, and then with the respective LA in three different concentrations: 0.1 mg/mL, 0.5 mg/mL, and then 1.0 mg/mL. To achieve optimal saturation of the uterus with oxygen, nutrients, and electrolytes, the initial perfusion was maintained for approximately 30 minutes. If rhythmic contractions were not observed, the experiment was stopped, and the respective uterus was excluded from data evaluation (see Fig 3).

### Intrauterine pressure measurement

Intrauterine pressure (IUP) was recorded with a probe consisting of a double-chip microcatheter (Urobar 8 DS-F, Raumedic Ltd., Rehau, Germany). The measurement sensors were incorporated into the catheter at intervals of 6.5 cm, so that after careful introduction, the distal sensor (IUP1) was positioned without tension in the one uterine horn and the proximal sensor in the body of the uterus (IUP2). The pressure catheter was then fixed with a single button suture at approximately 11 cm to prevent it from sliding out during the measurements. After this, the pressure catheter was connected with the monitoring device (Datalogger, MPR1, Raumedic Ltd., Rehau, Germany) using a connecting cable.

The data logger allowed simultaneous recording of pressure changes at both measurement points (IUP1, IUP2) and continuous temperature measurement. Pressure in mmHg was recorded by the Datalogger every second. Pressure conditions in the vascular and tubal system were displayed using a central venous pressure measurement module.

The probes named IUP1 and IUP2 and the central venous pressure were calibrated to 0 mmHg at the start of each experiment and the corresponding recording. The course of the experiment was observed on a graphic display and evaluated with the appropriate software (Datalogger; Raumedic).

### Data processing

For each uterus, data was analyzed in intervals of 10 min/20 min after the start of the experiment, for one hour. Evaluation of the data was carried out using the Origin program (OriginLab Northampton, Massachusetts, USA, version 8.5). Areas under the curve (AUC) were calculated. The AUC, as the total of all pressure values measured over a specific time interval, represented both the work performed by the myometrium and its output.

### Statistics

The data calculated by the Origin program were analyzed using Microsoft Excel and SPSS (IBM SPSS Statistics, version 21). Group comparisons were carried out after checking for normal distribution, using the Mann–Whitney *U* test and the Kruskal–Wallis test or one-way analysis of variance (significance level *p* = 0.05; post-hoc *p* = 0.017). In order to assess the number needed to treat for statistically significant differences between LA and their different concentrations, a sample size calculation was conducted. In order to achieve a significant difference of the AUC of 50, it was necessary to use 28 uteri per group at a given significance level of p<0.05 and a power β of 0.80.

## Results

### Study population

From 121 specimens in total, 91 uteri (75.2%) were included into the study evaluation. Exclusion criteria were: inadequate (low) temperature of the injection fluid (n = 14), insufficient uterine perfusion due to arterial vessel defects, probe defects (n = 6), or absence of uterine reactivity (i.e., no viability) before the beginning of the experiments (Fig 3). Mean weight was 137.6 grams (103–199 g). The following LAs were tested in three different concentrations each: procaine (n = 30 uteri), lidocaine (Lignocaine) (n = 30 uteri), and ropivacaine (n = 31 uteri).

### Influence of LAs in different concentrations

**Fig 4:**
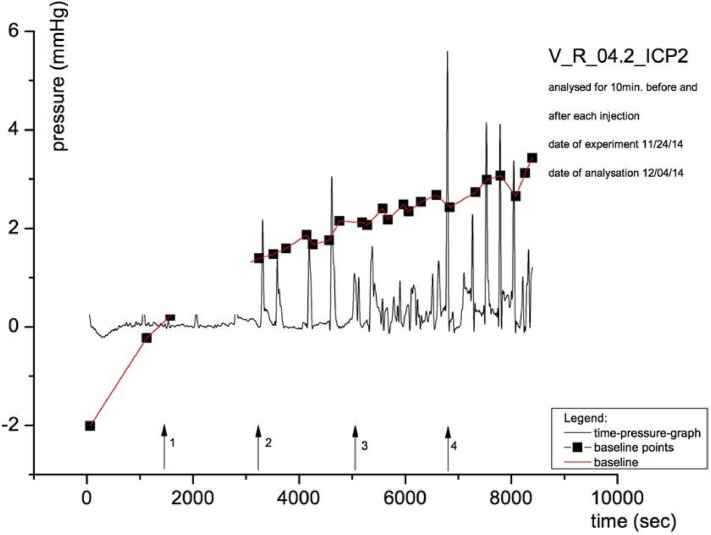
AUC of uterine contractility before and after application of KRB nutrient solution (arrow 1), 0.1% (arrow 2), 0.5% (arrow 3), and 1% (arrow 4) ropivacaine. KRB: Krebs-Ringer Buffer solution; y-axis: intrauterine pressure; x-axis: time in seconds.

A typical uterine pressure-time is shown for the LA ropivacaine in Fig 4.

#### Procaine

**Fig 5:**
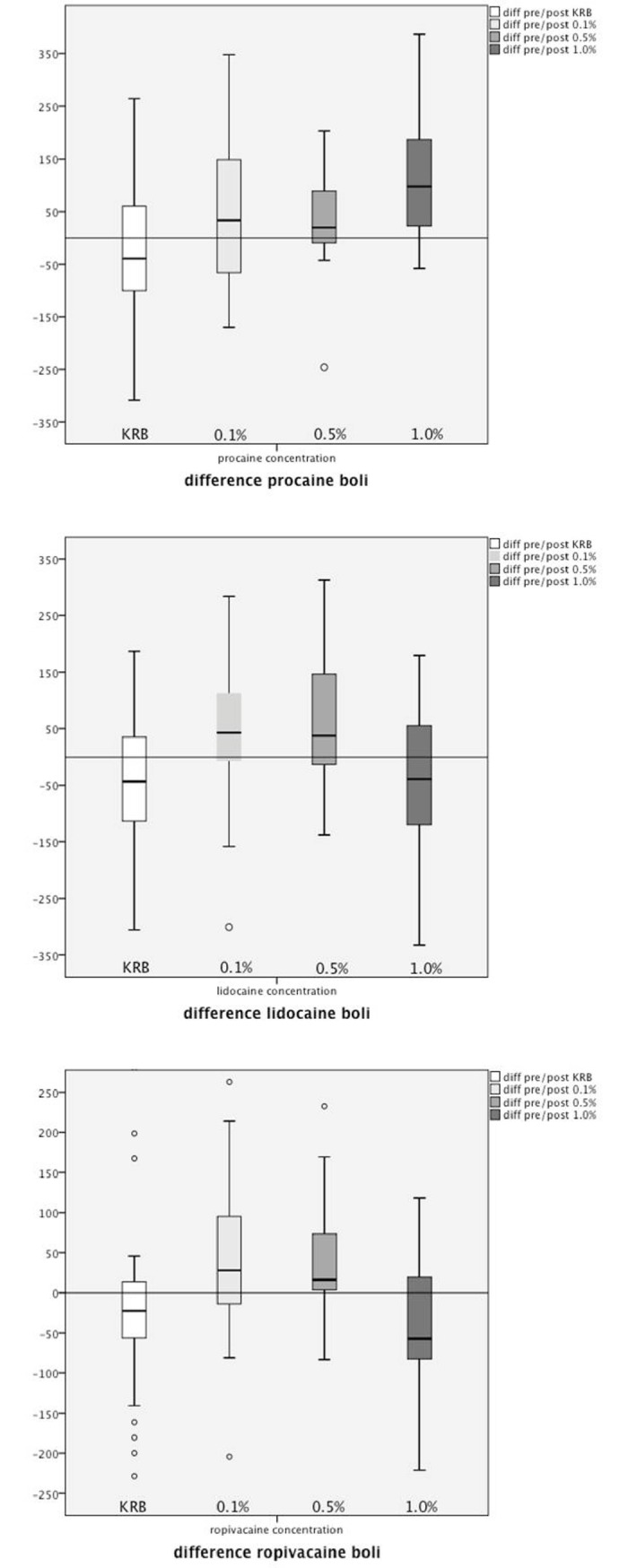
a-c. Change of uterine contractility after application of 0.1%, 0.5%, and 1% procaine (a), lidocaine (b), and ropivacaine (c). KRB: Krebs-Ringer buffer; boxplots: Pre-post-difference of the AUC after a bolus of 0.1%, 0.5%, and 1.0% of the respective LA. The y-axis is the change of AUC.

After uterus perfusion with procaine 0.1, 0.5 and 1.0%, a dose-dependent increase of contractility was observed. The differences between contractility after a bolus of buffer solution and 0.1% (p = 0,032), as well as between 0.5% and 1.0% (p = 0,013), and between a bolus of buffer solution and 1.0% (p<0,001) were significant (Fig 5A).

#### Lidocaine and ropivacaine

After perfusion with lidocaine in concentrations of 0.1, 0.5 and 1.0% an increase of contractility was seen for concentrations up to 0.5%. A decrease of contractility was observed with higher concentrations. The differences between the contractility after a bolus of buffer solution and of 0.1% lidocaine (p = 0,018) and ropivacaine (p = 0,015) were significant, as well as the difference between the contractility after 0.5% and 1.0% (p = 0,005) lidocaine and as after ropivacaine (p = 0,001) (Fig 5B and 5C).

### Difference of contractility patterns between LAs

**Fig 6:**
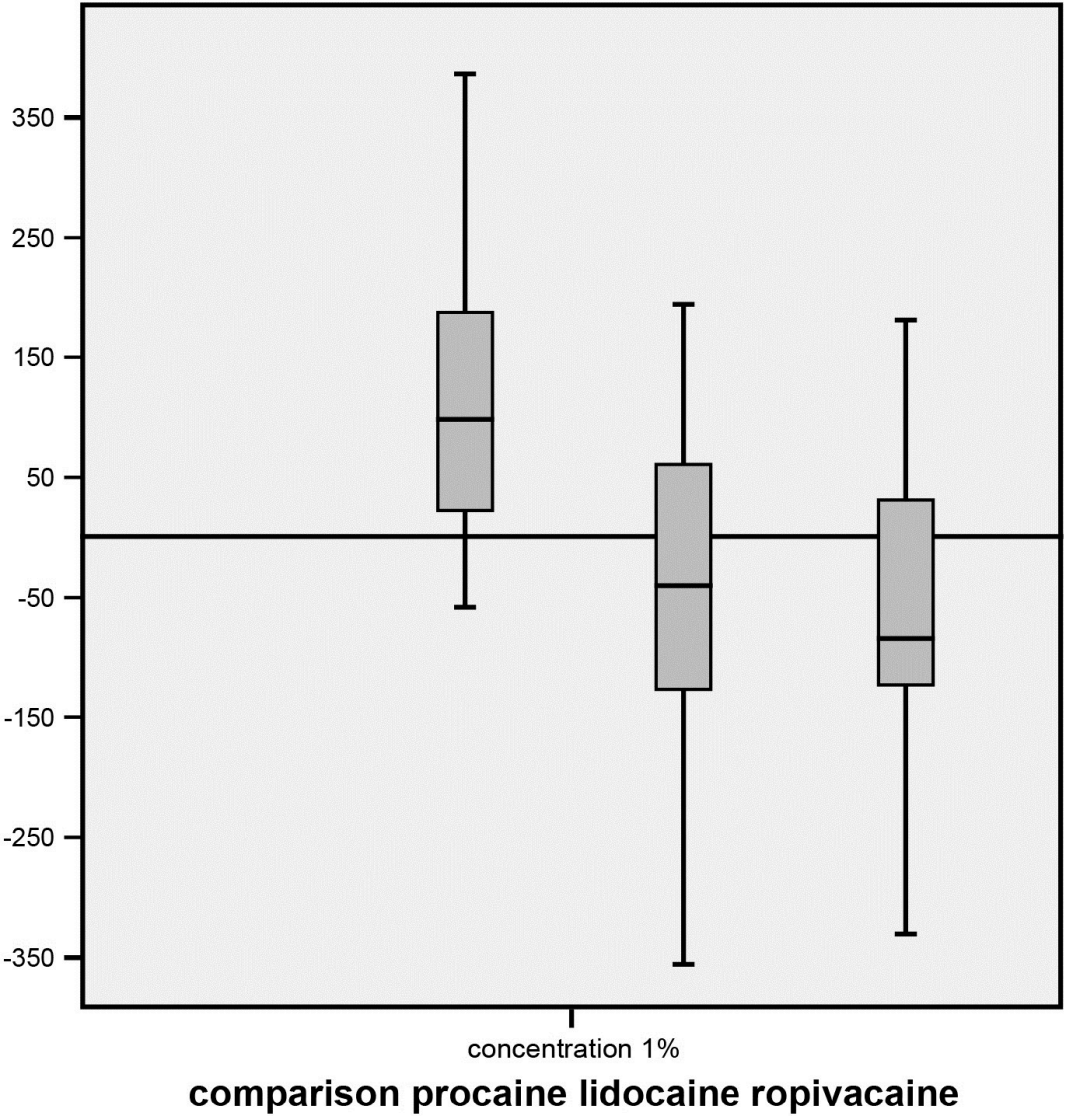
Comparison of the change of uterine contractility after application of 1.0% procaine, lidocaine, and ropivacaine, respectively. The differences between KRB and procaine (left), between procaine and lidocaine (middle), and between procaine and ropivacaine (right) are significant (p<0.05). The y-axis is the difference of AUC.

There was a significant difference of contractility (p<0.01) between ester-LAs and amide-LAs, which was observed at 1.0% concentrations. The difference between 1.0% lidocaine and 1.0% ropivacaine was not significant (Fig 6).

A summary of the study results is shown in Table 2.

**Table 2:** Change of uterine contractility by different LAs in different concentrations.

LA / concentration	Procaine	Lidocaine	Ropivacaine
0.1%	⇧	⇧	⇧
0.5%	⇧	⇧	⇧
1.0%	⇧	↓	↓

⇧ = increase, ↓ = decrease of contractility.

## Discussion

Dysfunction of contractility is an important factor in certain uterine disorders such as endometriosis [10], dysmenorrhea, and infertility [9]. LAs may be potent effectors on contractility through their pluripotent molecular mechanisms [20–21]. A number of contractility studies with LAs were performed in vitro. Most of them used isolated rat myometrial strips for the examination of LAs effects on myometrial tissue. However, they found contradictory results after LA application. Some of them described an increasing contractility of the uterus while others showed a decrease [22–24]. Other groups investigating isolated human myometrial strips also found contradictory results concerning myometrial contractility. Hurd et al. showed an increase of contractions after application of cocaine, an ester-linked LA [25]. In contrast, Fanning et al. investigating bupivacaine, fentanyl, other opiates, and oxytocin using the same model, described a decrease of amplitude and frequency of contractility after bupivacaine and levobupivacaine [26].

Another animal experiment performed *in vivo* was done injecting LAs into pregnant rat uteri. In this model, Kaynak et al. 2012 found a decrease of contraction frequency after administration of bupivacaine [27].

In contrast to the above-mentioned studies, this study demonstrates the effects of LAs on the contractility of the uterus *in vitro* in a whole-organ model of a perfused swine uterus. These are the first experiments conducted on a whole uterus model, perfused via the physiological path of arterial perfusion. This model is closer to the *in vivo* situation than isolated myometrial strips.

We used LAs in concentrations that are also used in daily clinical practice, and similar to those used in previous clinical studies [28]. We found that amide-linked LAs increased contractility at low concentrations but significantly decreased it in higher concentrations. In contrast, the ester-linked LA procaine significantly increased contractility with increasing concentrations. There was a significant difference between procaine and the two other LAs at high concentrations (~40 mmol/L).

In detail, we found partly activating effects: all three LAs, when administered in low concentrations of 0.1% and 0.5% compared to controls, induced an increase of uterine contractility. This effect was independent from the molecular structure of the respective LA. Nevertheless, lidocaine and ropivacaine in higher concentrations showed an inverse effect of relaxation of the uterine myometrium. As opposed to these two LAs, procaine, as the only ester-linked LA, showed a further increase of contractility when administered in higher concentrations (Table 2).

### Limitations and strengths of the study

Although the experiments were carried out in constant conditions, we found a high variability of uterine contractility between individual specimens. This phenomenon has also been observed in former experiments with this swine uteri model and raises the question whether the results presented in this study could be artifacts based on this high variability. High data variation could have been the result of inconstant placement of the intrauterine probe within the uterine lumen. Depending on whether or not the intrauterine probe was placed in direct contact with the uterine wall, different distances were measured every time the experiment was performed. This problem could be solved by placing a second probe in the other horn of the uterus and merging the results obtained from the two probes. However, because of the small size of the uterus compared to the diameter of the probe, it is challenging to actualize this adjustment.

Despite a high variation between individual specimen, we obtained significant results with a sufficient number of uteri. Therefore, we can safely exclude the influence of artifacts. The reason of the different effects caused by the three different LAs may in fact stem from their molecular structure.

The decrease in contractility in *high concentrations* of amide-LAs can be explained through a strong inhibition of the sodium channel of myocytes with subsequent relaxation of the muscle and decrease in contractility. However, the increase in contractility in *lower concentrations* of the amide-LA cannot be explained through this membrane effect.

Kasaba et al. described increasing neurotoxicity of LA *in vitro* in the following order: procaine < ropivacaine < lidocaine [29]. Therefore, myotoxic effects of amide-LAs may have been the main factor in decrease of contractility with higher dosages. Still, the increase with lower concentrations remains unexplained. Further studies with other amid-LA should elucidate this phenomenon.

As stated before, the molecular structure of the ester and amide-binding LAs may be the cause of this difference. Ester-LAs are degraded by local cholinesterase immediately after administration within the tissue. Nonetheless, if degradation of ester-LA procaine alone had played a role, we would have expected no change of contractility compared to KRB and the other LAs. To clarify this, further studies should also include other ester-LAs, such as chloroprocaine and tetracaine. The different behavior of procaine might be attributed to the pharmacological effects of its cleavage products. Procaine is cleaved into the two metabolites diethylaminoethanol (DEAE) and para-aminobenzoic acid (PABA), each with specific pharmacological effects, e.g., DEAE has a vasodilatory effect [30].

Interactions with other receptor proteins could also explain this different behavior. Under these receptor proteins we find: calcium channels [1], potassium channels, NMDA receptors [31] and TRPV1 (capsaicin receptor) [32]. Further experiments with receptor blocking agents will clarify the exact impact of these receptors.

### Clinical relevance for reproductive medicine

Auto-traumatization by uterine peristalsis and hyper-peristalsis is an important mechanism for the development of adenomyosis and endometriosis [12]. The treatment of abnormal peristalsis with LAs is a promising indication that requires further studies.

Unexplained infertility is another promising field of LAs application. Future studies could demonstrate an increased fertility rate after intrauterine instillation of lidocaine [9]. The relaxation of the uterus as shown in our survey–resulting particularly from administration of amide-LAs as lidocaine in higher concentrations–may explain these clinical results.

Taken together, we conclude that LAs are potent effectors on uterine contractility. Perhaps, amide-LAs will be more suitable for disorders with increased uterine contractility, whereas ester-LAs as procaine may be helpful in situations where a stimulation of intrauterine contractility may be desired. Further studies with other LAs in different concentrations will clarify these questions.

## Supporting information

S1 Dataset Database of 91 uteri and their contractility changes. (ZIP)
